# Aging-related deficits accumulation and cancer-related outcomes in testicular and prostate cancer: Cross-sectional and longitudinal findings

**DOI:** 10.1016/j.socscimed.2026.119332

**Published:** 2026-04-24

**Authors:** Michael A. Hoyt, Karen Llave, Judith E. Carroll, Marcie D. Haydon

**Affiliations:** a Department of Population Health and Disease Prevention, Joe C. Wen School of Population & Public Health, University of California, Irvine, 856 Health Sciences Road, Suite 3800, Irvine, CA, 92617-9937, USA; b Chao Family Comprehensive Cancer Center, University of California, Irvine, 200 S Manchester Road, Orange, CA, 92868, USA; c Center for Biomarker & Biobehavioral Research, University of California, Irvine, 856 Health Sciences Road, Irvine, CA, 92617-9937, USA; d Department of Psychiatry and Biobehavioral Sciences, University of California, Los Angeles, 760 Westwood Plaza, Los Angeles, CA, 90095, USA

**Keywords:** Aging, Survivorship, Deficit accumulation index, Testicular cancer, Prostate cancer, Inflammation

## Abstract

This study examined how aging-related deficit burden relates to cancer-specific outcomes in men diagnosed at markedly different developmental periods and with different cancer experiences. A modified Deficit Accumulation Index (DAI), a multi-system measure of aging based on proportional health deficits, was computed in two cohorts. Study 1 used cross-sectional data from 171 young adult testicular cancer survivors (ages 18–29). Study 2 used longitudinal data from 114 men with localized prostate cancer (ages 51–81) assessed before treatment and six months post-treatment.

In Study 1, DAI scores ranged from 0 to .73 (M = .23), with 47.7% of young adult participants exhibiting medium or high deficit burden. Higher DAI was associated with greater job problems, family disruption, sexual problems, and treatment side effects after adjusting for age and time since diagnosis (p’s < .001). In Study 2, DAI scores ranged from 0 to .55 (M = .18), with 34.0% exhibiting medium or high deficit burden. Baseline DAI predicted worsening in hormonal functioning six months after treatment. Greater DAI was also associated with higher circulating interleukin-6 prior to treatment (p < .01). Across both cohorts, deficit accumulation functioned as a marker of survivorship burden; however, the specific outcomes associated with higher deficit burden differed across cohorts. Findings demonstrate the utility of the DAI as a multi-system metric for understanding vulnerability in cancer survivorship.

## 1. Background

The biological, clinical, and psychosocial phenomena of cancer and aging are deeply intertwined. Yet survivorship science has only recently begun to conceptualize cancer as a potential driver of premature aging (Guida et al., 2019; New York Academy of Sciences, 2016; Wang et al., 2021). Although aging is traditionally considered within the context of older adulthood, geroscience frameworks underscore that aging is the accumulation of multi-system physiological deficits that gradually degrade reserve and resilience, and render individuals more susceptible to morbidity and functional impairment (Mitnitski and Rockwood, 2015). This understanding has prompted a paradigm shift in which cancer and its treatments are viewed as physiological stressors that can facilitate more rapid biological and functional aging, often years to decades earlier than expected for chronological age (Bhatia et al., 2022; Carroll et al., 2022; Kruk et al., 2019).

In fact, premature aging has important life-course implications, as cancer and its treatments may initiate early physiological decline that accrues clinical significance over long post-treatment timelines, particularly among younger men diagnosed with testicular cancer, which is the most common malignancy in males aged 15–39 (Chovanec et al., 2021; Fosså et al., 2025; Gehle et al., 2023; Henderson et al., 2014). On the other hand, older men, who account for most prostate cancer diagnoses, often enter treatment with age-related diminished physiological reserve, rendering them particularly vulnerable to cancer- and treatment-related stressors that may accelerate biological and functional aging processes (Sedrak et al., 2021). A lifespan lens may provide important theoretical precision (Mandelblatt et al., 2021). The timing of cancer within the life course may shape not only the detectability of premature aging, but also its psychosocial meaning, functional burden, and long-term clinical consequences (Henderson et al., 2014).

Testicular cancer survivorship offers a compelling illustration of how premature aging can unfold across the life course. Testicular cancer is typically diagnosed in young adulthood and is highly curable, with survival rates exceeding 95% for localized disease. Standard treatments are effective but biologically consequential, inducing DNA damage, oxidative stress, vascular injury, and endocrine disruption that are themselves implicated in long-term functional decline and the emergence of aging-related conditions such as sarcopenia, hypogonadism, and cardiometabolic disease (de Haas et al., 2013; Fung et al., 2019; Haugnes et al., 2010). Because most survivors live for many decades after treatment, even subtle early health deficits can gradually accumulate over time, potentially shaping trajectories of chronic disease as well as development of secondary cancers (van der Meer et al., 2024). Importantly, this biological wear and tear unfolds alongside key developmental tasks of early adulthood including establishing careers, forming long-term partnerships, making fertility decisions, and consolidating personal identity. This confluence creates a distinct intersection between aging processes and psychosocial development, which is rarely considered in survivorship care.

In contrast, prostate cancer survivorship is predominantly situated in later adulthood, a period already characterized by gradual physiological reserve loss and increasing vulnerability to chronic illness. As a result, the field has increasingly turned to aging and gerontological frameworks to understand survivorship trajectories, given the substantial overlap between prostate cancer treatment effects and geriatric susceptibility (Han et al., 2025). Common systemic therapies such as androgen deprivation therapy intentionally suppress testosterone, which accelerates loss of lean muscle mass, reduces bone density, and disrupts metabolic regulation while heightening fatigue. These treatment-related changes intersect with normative biological aging processes, potentially accelerating transitions from independence to frailty. For many men, this can translate into meaningful declines in mobility, elevated fall risk, and threats to autonomy at a stage of life when maintaining functional independence is central to wellbeing (Bylow et al., 2007; Park et al., 2023). Prostate cancer survivorship thus represents a convergence of adverse cancer treatment impact and late-life aging, where medical interventions may reshape not only health trajectories but also social roles, caregiving needs, and quality of life in advanced adulthood.

Aging in cancer survivorship is not simply a matter of chronological time. Rather, it reflects the cumulative imprint of biological stressors, disease exposures, and social context unfolding across decades. In this context, the Deficit Accumulation Index (DAI) offers a useful framework for conceptualizing and quantifying aging in oncology. Rather than relying on a single biomarker or organ-specific pathology, the DAI operationalizes aging as the proportional accumulation of impairments across multiple physiological and functional systems, including physical performance, cognition, sensory function, metabolic health, comorbid disease, and limitations in daily activity (Mitnitski et al., 2001; Williams et al., 2023).

A growing body of evidence demonstrates that the DAI robustly predicts morbidity, mortality, hospitalization, disability, and functional decline, including among cancer survivors. For example, a recent large analysis demonstrated that higher DAI scores were associated with increased mortality risk and accelerated aging phenotypes even without specific molecular biomarkers, supporting the validity of the DAI as an aging metric among long-term cancer survivors (Williams et al., 2023). This aligns with earlier foundational work establishing that deficit accumulation models outperform simple comorbidity counts in forecasting adverse aging outcomes (Mitnitski et al., 2001; Rockwood et al., 2004). Importantly for oncology, the DAI has been used to capture treatment-related aging acceleration, enabling researchers to characterize “off-time” aging in younger survivors and “compressed-reserve” aging in older adults with cancer. Recent conceptual work further argues that the DAI is uniquely well suited for cancer research because it integrates the multi-system biological consequences of treatment with the social and behavioral dimensions of aging across the life course (Mohile et al., 2018).

Building on this conceptual foundation, the present paper leverages data from two studies of men with cancer by computing DAI to examine relationships of premature aging and cancer-relevant biomarkers and health indicators across distinct developmental contexts and differing cancer treatment profiles. By focusing on younger men treated for testicular cancer and older men treated for prostate cancer, we examine how deficit accumulation relates to physiological vulnerability and functional outcomes across two cohorts that differ in life-course timing, treatment exposures, and clinical context. In Study 1, we use cross-sectional data from young adult testicular cancer survivors to characterize associations of deficit accumulation with cancer-related occupational, family, and sexual problems. In Study 2, we employed longitudinal data across the 6-month post-treatment period from men with prostate cancer to evaluate how deficit accumulation at baseline is associated with changes in prostate cancer-specific functional outcomes over the post-treatment phase (e.g., 6 months after treatment). Consistent with observational study conventions, we did not define primary and secondary outcomes. We also explored associations between deficit accumulation and inflammation in the pre-treatment phase. Together, these complementary analyses aim to advance a developmentally-informed understanding of aging in oncology, while demonstrating the utility of the DAI as a multi-system metric of survivorship aging.

## 2. Study 1

### 2.1. Method

#### 2.1.1. Procedures

Eligible individuals were identified through the California Cancer Registry and were invited to participate in a study of cancer survivorship in young adults treated for testicular cancer. Eligible individuals were between the ages of 18 and 29, with a confirmed history of testicular cancer, and the ability to read and understand English (see Hoyt et al., 2013). Men were excluded if they reported a history of severe psychiatric disorder or significant cognitive dysfunction. Participants underwent informed consent procedures and completed study assessments for which they were given $50. Study procedures were approved by the human subjects’ protection boards at the University of California and the State of California Committee for the Protection of Human Subjects (CPHS approval number: 12-05-0271).

#### 2.1.2. Measures

##### Deficit Accumulation Index (DAI).

Biological and functional aging was operationalized using the DAI, consistent with established frailty and aging frameworks but tailored to the measures available in the present dataset. The DAI was constructed using 32 aging-related indicators meeting established DAI criteria derived from participant self-report measures, as well as cancer registry and medical record data (see Supplemental Table 1) (Rockwood et al., 2006). Items spanned multiple physiological and psychosocial domains, including chronic health conditions, functional limitations, psychosocial functioning, and mental well-being. DAI items did not include any of the outcome variables used in regression models.

Each indicator was coded on a scale from 0 to 1, with higher values reflecting greater presence or severity of a deficit. Item scores were summed and divided by the total number of available items to generate a continuous index ranging from 0 to 1, with higher scores indicating greater deficit accumulation and reduced physiological reserve to withstand future stressors as outlined by Williams and colleagues (2023) in survivors of childhood cancers. To enhance interpretability and facilitate comparison with prior studies, DAI scores were also classified into clinically meaningful categories: low (or robust) (0 to <0.2), medium (or pre-frail) (0.2 to <0.35), and high (frail) (≥0.35), corresponding to thresholds previously associated with increased risk of hospitalization and mortality (Rockwood and Howlett, 2018; Searle et al., 2008).

##### Cancer-related problems.

To assess testicular cancer-specific occupational, family, and sexual problems participants completed the testicular cancer module of the EORTC-QLQ-C30 (Fosså et al., 2003). These included the job problems (“Have you had any problems with your job or your education because of your disease or treatment?”), family problems (“Were you concerned about disruption of family life?), and sexual problems (ejaculatory and erectile function) scales. The eight-item treatment side effects scale assessing frequency of common treatment-related effects (e.g., hair loss, hearing problems) was also administered. All items were rated on a 4-point scale from 1 (not at all) to 4 (very much). Scoring was performed according to established conventions where a higher score represents a worse outcome (Fosså et al., 2003).

##### Demographic and clinical information.

Demographic and clinical data, including body mass index (BMI) and testicular cancer-related treatment information, were assessed via medical record review and via self-report. In addition, medical co-morbidities were recorded.

#### 2.1.3. Data analysis

Descriptive statistics were computed for key study variables, including sociodemographic and relevant clinical characteristics. To further examine age acceleration, resulting residuals from regressing the DAI on chronological age were examined. Positive residuals were considered to indicate greater deficit burden than expected for chronological age within the present sample, reflecting relatively premature aging compared to other cohort members (Crimmins et al., 2021).

Hierarchical linear regression in SPSS statistical software (version 28; SPSS, Chicago, IL, USA) was used to examine relationships job, sexual, and family problems as well as treatment side effects with DAI. Regression models were organized in two steps in which the statistical covariates were entered in the first step, and DAI scores in the second step. Participant age, time since diagnosis, and income were identified as statistical covariates and included in regression models. Income was included as a covariate as a proxy for socioeconomic status, given its established associations with health outcomes and biological aging processes (Steptoe and Zanotto, 2020). This approach allowed for the examination of the incremental contribution of DAI over and above covariates.

### 2.2. Results

#### 2.2.1. Participants

In total, 171 young adults (mean age = 25.2, standard deviation = 3.32, range = 18-29) completed study assessments. Participant characteristics are reported in Table 1. Most were single or unmarried, and a majority having earned at least a high school level of education. Approximately 53% of participants identified as an ethnic or racial minority. The average time since diagnosis was 32.4 months (standard deviation = 19.3). Most participants underwent surgical intervention including orchiectomy (80.1%) and retroperitoneal lymph node dissection surgery (24%); 53.2% received chemotherapy and 15.2% received radiation therapy. They self-reported diagnoses at stage I (34.5%), stage II (18.1%), and stage III (12.9%); however, 34.5% did not know their diagnostic stage.

#### 2.2.2. Accelerated aging

DAI scores ranged from 0 to .73 (mean = .23, standard deviation= .16). Most participants (52.3%) were categorized as low or robust, 31.4% medium or pre-frail, and 16.3% as high or frail. To signal age-related deviation in biological aging, we computed age-adjusted DAI residuals by regressing DAI on chronological age (Crimmins et al., 2021). Positive residuals were interpreted as indicating a higher deficit burden than expected for chronological age (i.e., accelerated aging), whereas negative residuals reflected fewer deficits than expected (i.e., decelerated aging). Exactly 42.1% showed positive residuals indicating greater than expected deficit burden for age, whereas 57.9% demonstrated negative residuals (decelerated aging). The magnitude of age acceleration ranged from − .23 to .50.

#### 2.2.3. Associations with survivorship outcomes

Participants reported job problems (mean = 20.49; standard deviation = 29.29), family problems (mean = 25.88, standard deviation = 32.38), sexual problems (mean = 12.58, standard deviation = 21.77), and treatment side effects (mean = 14.15, standard deviation = 16.79).

We examined the relationships of DAI scores with each outcome of interest, adjusting for age and time since diagnosis (see Table 2). DAI scores were positively associated with job problems (standardized regression coefficient = .69, probability <.001), family problems (standardized regression coefficient = .49, probability <.001), sexual problems (standardized regression coefficient = .45, probability <.001), and treatment side effects (standardized regression coefficient = .70, probability <.001). After adjusting for age and time since diagnosis, the DAI accounted for 18-44% of the variance cancer outcomes.

## 3. Study 2

### 3.1. Method

#### 3.1.1. Procedures

English-speaking men choosing to undergo radical prostatectomy or radiation therapy for localized prostate cancer were recruited to take part in a larger longitudinal study of health-related quality of life after prostate cancer. Men with a lifetime history of severe mental illness or substantial cognitive impairment were excluded. A subset of participants provided blood sample for assessment of biomarkers. Men with medical conditions, medication use, or frequent substance use were excluded from biomarker assessment.

The Institutional Review Boards at the University of California approved all procedures. After providing written informed consent, participants completed baseline assessments including a questionnaire, interview, and a blood draw for inflammation assessment. Baseline procedures occurred prior initiation of prostate cancer treatment. Participants repeated all assessments at 6-months post-completion of primary treatment. Each received $25 for completing questionnaires at baseline and at 6-months post-treatment ($50 total).

#### 3.1.2. Measures

##### Deficit Accumulation Index (DAI).

Biological and functional aging was operationalized using an adapted DAI similar to Study 1. The DAI variable for Study 2 was constructed using 34 of the standard aging-related indicators derived from participant self-report measures and medical record data (see Supplemental Table 1).

##### Prostate-specific function.

To assess prostate cancer-specific functioning, participants completed the Expanded Prostate cancer Index Composite (EPIC; Wei et al., 2000). EPIC measures disease-specific aspects of prostate cancer and treatment. It is comprised of a urinary, bowel, sexual, and hormonal domains. EPIC item responses form a Likert scale and domain scores are transformed on a linear 0-100 scale. Higher scores indicate more favorable functioning.

##### Pro-inflammatory biomarkers.

Blood samples for measurement of circulating *pro-inflammatory markers* were collected via venipuncture into EDTA tubes, placed on ice, centrifuged at 4 °C for 15 min for harvesting of plasma within 30 min, and stored at − 80 °C until subsequent batch testing. Five biomarkers that have pro-inflammatory properties (interleukin-6 [IL-6], C-reactive protein [CRP], soluble tumor necrosis factor receptor II [sTNFαRII]), interleukin-1 beta [IL-1β], interleukin-8 [IL-8]) were measured. All plasma samples were run in duplicate using procedures recommended by the assay manufacturer; for MesoScale Discovery (MSD [Rockville, MD]) assays, each biomarker was assayed in multi-plex. Higher values indicate more overall circulating levels of the respective biomarker.

##### Demographic and clinical information.

Demographic and clinical data, including body mass index (BMI) and medical co-morbidities were assessed via medical record review and self-report.

#### 3.1.3. Data analysis

Descriptive statistics were computed for key study variables including sociodemographic and relevant clinical characteristics. Again, to examine age acceleration, resulting residuals from regressing the DAI on chronological age were examined. Positive residuals were considered to indicate greater deficit burden than expected for age and thus accelerated aging relative to peers.

Hierarchical linear regression in SPSS statistical software (version 28; SPSS, Chicago, IL, USA) was used to examine urinary, bowel, hormonal, and sexual function as well as pro-inflammatory markers with DAI. Regression models were organized in two steps in which the statistical covariates were entered in the first step, and DAI scores in the second step. Participant age, income, and time since diagnosis were identified as statistical covariates and included in regression models (see O’Connor et al., 2009). To focus on baseline to 6-month change in function, baseline function scores were entered in all models.

### 3.2. Results

#### 3.2.1. Participants

Participants (N = 114) ranged in age from 51 to 81 years (mean = 65.58, standard deviation= 6.22) and were recruited from public hospitals (N = 20), NCI-designated comprehensive cancer center (N = 74), and a large urban Veteran’s Affairs medical center (N = 20). The average time since diagnosis at study entry was 10.73 months (standard deviation = 34.22; Range = <1 to 305). Among the participants, 93% received radical prostatectomy and 7% received radiation therapy. Additional clinical and socio-demographic variables are reported in Table 1.

At baseline, participants reported a mean of 71.25 for urinary (standard deviation = 13.24), 81.47 for bowel (standard deviation = 8.87), 56.32 for sexual (standard deviation = 22.81), and 76.15 for hormonal (standard deviation = 10.91) functioning. Across domain, functioning declined by 6-months post-treatment for urinary (mean = 68.53, standard deviation = 13.21), bowel (mean = 81.77, standard deviation = 10.25), sexual (mean = 34.58, standard deviation = 20.95), and hormonal (mean = 76.02, standard deviation = 9.80).

The biomarker subsample consisted of 37 participants ages 51 to 74 years (mean = 62.51, standard deviation = 5.49). On average, the subsample was 6.29 months from diagnosis at study entry (standard deviation = 18.55; Range = <1 to 106). Majority of participants in the biomarker subsample received radical prostatectomy (N = 34, *91.9%*) and the average BMI for this subsample was 28.22 (standard deviation = 4.94) at study entry.

#### 3.2.2. Accelerated aging

DAI scores ranged from 0 to .55 (mean = .18, standard deviation = .10). Most participants (66.0%) were categorized as low or robust, 29.9% medium or pre-frail, and 7.1% as high or frail. When examining age-adjusted DAI residuals, 35.4% of participants exhibited positive residuals (accelerated aging), whereas 64.6% demonstrated negative residuals (decelerated aging). The magnitude of age acceleration ranged from −.16 to .32.

#### 3.2.3. Associations with prostate functioning

At baseline, DAI scores were associated with worse baseline function across domains: urinary (correlation coefficient = −.48, probability< .001), bowel (correlation coefficient = −.49, probability< .001), sexual (correlation coefficient = −.35, probability< .001), and hormonal (correlation coefficient = −.48, probability < .001) functioning.

Regression analyses predicting changes in functioning across the 6-month post-treatment period adjusted for age, income, time since diagnosis, as well as pre-treatment function scores (see Table 3). DAI scores predicted worsening in hormonal functioning (standardized regression coefficient = −.35, probability< .001) and accounted for 8.4% of the variance in hormonal function at 6-months post-treatment. However, DAI was not significantly predictive of changes in urinary, sexual, or bowel functioning.

#### 3.2.4. Associations with inflammation biomarkers

Associations of DAI and inflammation markers are reported in Table 4. After adjusting for age, income, and time since diagnosis, greater accumulated deficit was associated with more circulating IL-6 (standardized regression coefficient = .53, probability< .01), but not CRP, sTNFαRII, IL-1β, or IL-8.

## 4. Discussion

This study focused on premature aging in cancer survivorship by applying a deficit accumulation framework to two male cancer populations located at markedly different points in the lifespan. Integrating observations from young adult testicular cancer survivors with data from older men treated for prostate cancer, results support the possibility that cancer and its treatments promote aging marked by accumulation of deficits. Notably, nearly half of young adult survivors fell into DAI categories typically observed in much older community populations (Mitnitski et al., 2001; Rockwood et al., 2004), suggesting the presence of a premature aging phenotype despite much younger chronological age. Deficit accumulation operates as a meaningful marker of survivorship burden in both groups. At the same time, the interpretation, clinical significance, and intervention implications of this deficit burden likely differs across cohorts, potentially reflecting differences in life course timing, treatment exposures, and differences in the assessment of deficit burden itself. In both studies, the DAI was associated with cancer-specific functional problems; however, the implications of these deficits for survivorship care may differ.

In young adult testicular cancer survivors, higher DAI scores were associated with a set of salient domains of survivorship burden including occupational problems, family disruption, sexual dysfunction, and treatment side effects. In older prostate cancer patients, baseline DAI predicted declines in treatment-related hormonal function six months after treatment. Although the DAI was not uniformly related to the measured inflammation markers, DAI scores in the prostate cohort was associated with circulating IL-6, a biomarker central to the biology of aging and frailty (Ferrucci and Fabbri, 2018; Zhang et al., 2023). These converging findings align with geroscience perspectives that conceptualize aging not as chronological time but as cumulative multi-system vulnerability that erodes reserve and resilience (Mitnitski et al., 2001; Mitnitski and Rockwood, 2015).

For young adult men with testicular cancer, deficit accumulation appears to reflect the imprint of cancer and its treatment on aging trajectories that might otherwise be expected to be minimal at this stage of life. Nearly half of participants demonstrated age-accelerated DAI residuals, indicating greater deficit burden. The strong associations between DAI and disruptions in work, family life, sexual function, and treatment side effects suggest a form of “off-time” aging, in which physiological deficits manifest during a period characterized by consolidation of occupational identity, formation of long-term partnerships, fertility decisions, and identity development (Henderson et al., 2014). The psychosocial salience of these deficits is amplified because they occur in young adulthood, a period when physical vitality and functional independence are normative expectations. Here, deficit burden is embedded within developmental role expectations and life goal trajectories in ways that may reverberate across decades.

In contrast, men in the prostate cancer cohort likely meet diagnosis with a higher baseline deficit burden due to normative aging processes, co-morbidities, and pre-treatment physiological status that have gradually eroded physiological reserve and may have made them more vulnerable to development of cancer (Sedrak et al., 2021). Cancer treatment, particularly androgen deprivation therapy and prostatectomy or radiation therapy, might intersect with this reduced reserve functioning as a two hit model, by increasing vulnerability to additional aging by promoting slow or incomplete recovery, endocrine dysfunction, and loss of independence (Bylow et al., 2007; Park et al., 2023). Thus, the DAI may serve as a practical tool for informing treatment decisions for the individual patient rather than relying on biological age alone. Geriatric oncology guidelines increasingly emphasize frailty and functional status when considering treatment options (Skolarus et al., 2014). The DAI reframes the clinical question from one of chronological age to one of physiological reserve. For patients with high deficit burden, the risks of more aggressive treatments may outweigh potential benefits, and greater emphasis may be given to supportive care, rehabilitation, and maintaining independence.

For young adults, implications may differ. Here, the DAI may identify individuals at risk for longer-term aging-related morbidity long before it becomes clinically visible. The associations between DAI and psychosocial and functional problems suggest that deficit burden is already shaping survivorship experiences in early adulthood. This suggests a critical window for preventive intervention before deficit trajectories become clinically entrenched (see Mandelblatt et al., 2021). Interventions targeting physical activity, muscle preservation, endocrine and metabolic regulation, and psychosocial coping may be particularly important (Carroll et al., 2022).

The biomarker findings provide insight into the biological underpinnings of deficit accumulation. Results show a selective association between DAI and IL-6, but not CRP, sTNFRII, IL-1β, or IL-8. IL-6 is widely recognized as a biomarker of a chronic low-grade inflammatory state associated with frailty, endocrine disruption, and multi-system decline (Ferrucci and Fabbri, 2018; Franceschi et al., 2000). Elevated IL-6 has repeatedly been linked to disability, mortality, and deficit accumulation in older adults (Hubbard et al., 2009). The selective association with IL-6 reinforces the interpretation that the DAI is capturing a systemic aging phenotype rather than general inflammation. This finding supports the notion that cancer-related aging processes may operate through endocrine-immune-metabolic pathways uniquely indexed by IL-6.

Importantly, differences observed across cohorts should be interpreted with consideration of methodological and clinical differences. The testicular cancer cohort was assessed approximately three years post-treatment and included substantial exposure to systemic chemotherapy, whereas the prostate cancer cohort was assessed prior to treatment and primarily received localized treatments. These differences in treatment modality and timing are likely to influence deficit accumulation and survivorship outcomes. As such, observed differences between cohorts cannot be attributed solely to biological age differences or developmental life stage. Rather, they likely reflect a combination of developmental context, treatment-related late effects, and differences in assessment strategies. Future research will be necessary to more directly disentangle these influences.

Several additional considerations warrant attention. First, the DAI in both studies was constructed retrospectively using available measures rather than being prospectively designed as an aging index. Relatedly, because these datasets were not originally designed to assess aging processes, the breadth and weighting of included indicators were constrained by available variables, potentially influencing the precision with which deficit burden was captured. In addition, there were modest differences in the number and type of items that composed the DAI across cohorts (see Supplementary Table 1). While the deficit accumulation framework is designed to accommodate heterogeneity in indicators and capture clinically meaningful burden regardless of specific item content, these differences could have contributed to variation in pre-frailty and frailty. Further, differences in time since treatment and treatment type may also have influenced deficit profiles. These factors should be considered when interpreting cross-cohort comparisons.

Notably, sample sizes in both cohorts were modest, which may have limited statistical power and the ability to conduct subgroup analyses that could further clarify how deficit accumulation operates across heterogeneous survivorship experiences. Also, the cross-sectional nature of some analyses limits causal inference regarding aging trajectories. Biomarkers were available only in the prostate cancer cohort and only at baseline. This precluded examination of how inflammatory processes change across the treatment trajectory or how changes in inflammation relate to evolving deficit burden. Additionally, reliance on a limited panel of inflammatory markers restricts interpretation of the broader biological pathways through which deficit accumulation may operate. Finally, the use of existing datasets limited the ability to include molecular markers of aging, performance-based physical function measures, and objective assessments of physiological reserve. Future studies designed specifically to examine aging in cancer survivorship and incorporating prospective deficit measurement, larger samples, repeated biomarker assessments, and molecular aging indicators will be essential for more precisely characterizing how deficit accumulation develops and interacts with cancer treatment across the life course.

In conclusion, these findings demonstrate that deficit accumulation provides a powerful framework for understanding how aging and cancer intersect across the life course. For young men with testicular cancer, cancer and its treatments may contribute premature aging processes with long psychosocial and biological consequences. For older men with prostate cancer, pre-existing aging processes, along with baseline health status, may shape vulnerability to treatment and functional decline. Recognizing differences in deficit burden across these distinct survivorship contexts may help inform more precise survivorship care, more thoughtful treatment decision-making, and targeted interventions designed not only to manage cancer outcomes but also to alter aging trajectories in meaningful ways.

## Supplementary Material

Supplementary Table 1

## Figures and Tables

**Table 1:** Participant characteristics.

Characteristic	Study 1: Testicular Cancer				Study 2: Prostate Cancer			
	Total Sample (N = 171)	Low DAI (n =89)	Medium DAI (n = 54)	High DAI (n =28)	Total Sample (N = 114)	Low DAI (n = 75)	Medium DAI (n =33)	High DAI (n = 6)
	n (%)	n (%)	n (%)	n (%)	n (%)	n (%)	n (%)	n (%)
Age	25.2 (SD = 3.32)	25.2 (SD = 3.20)	25.1 (SD = 3.39)	25.4 (SD = 3.67)	65.6 (*SD* = 6.22)	64.9 (SD = 5.56)	67.0 (SD = 6.77)	65.4 (SD = 10.33)
Ethnicity								
White (non-Hispanic)	79 (46.2)	45 (50.6)	22 (40.7)	12 (42.9)	63 (55.3)	41 (59.4)	18 (56.3)	4 (66.7)
Hispanic/Latino	65 (38.0)	31 (34.8)	23 (42.6)	11 (39.3)	10 (8.8)	8 (11.6)	1 (3.1)	1 (16.7)
Asian	18 (10.5)	8 (9.0)	7 (13.0)	3 (10.7)	21 (18.4)	6 (8.7)	3 (9.4)	1 (16.7)
Native American/Alaskan Native	5 (2.9)	3 (3.4)	0 (0.0)	2 (7.1)	0 (0.0)	0 (0.0)	0 (0.0)	0 (0)
African American/Black	2 (1.2)	1 (1.1)	1 (1.9)	0 (0.0)	10 (8.8)	13 (18.8)	8 (25.0)	0 (0)
Other	2 (1.2)	1 (1.1)	1 (1.9)	0 (0.0)	6 (5.3)	4 (5.8)	2 (6.3)	0 (0)
Education								
Less than High School	8 (4.7)	4 (4.5)	1 (1.9)	3 (10.7)	8 (7.0)	6 (8.6)	2 (6.2)	0 (0.0)
High School/GED	26 (15.2)	10 (11.2)	9 (16.7)	7 (25.0)	23 (20.2)	9 (12.8)	12 (37.6)	2 (33.4)
Some College	55 (32.2)	20 (22.5)	24 (44.4)	11 (39.3)	19 (6.7)	10 (14.3)	6 (18.8)	3 (50.0)
2-year College Degree	19 (11.1)	13 (14.6)	3 (5.6)	3 (10.7)	10 (8.8)	6 (8.6)	4 (12.5)	0 (0.0)
4-Year College Degree	47 (27.4)	31 (34.8)	12 (22.3)	4 (14.3)	20 (17.5)	16 (22.9)	3 (9.4)	0 (0.0)
Graduate Degree	16 (9.4)	11 (12.4)	5 (9.3)	1 (3.6)	28 (24.6)	22 (31.5)	5 (15.7)	1 (16.7)
Income								
$15,000 or less	41 (24.0)	13 (14.6)	16 (29.6)	12 (42.9)	10 (8.8)	7 (10.1)	3 (9.4)	0 (0.0)
$15,001-$30,000	34 (19.9)	17 (19.1)	12 (22.2)	4 (14.3)	12 (10.5)	5 (7.2)	5 (15.6)	2 (33.3)
$30,001-$45,000	20 (11.7)	7 (7.9)	9 (16.7)	4 (14.3)	4 (3.5)	4 (5.8)	0 (0.0)	0 (0.0)
$45,001-$60,000	26 (15.2)	18 (20.2)	7 (13.0)	1 (3.6)	10 (8.8)	4 (5.8)	6 (18.8)	0 (0.0)
$60,001-$75,000	19 (11.1)	10 (11.2)	4 (7.4)	5 (17.9)	8 (7.0)	3 (4.3)	3 (9.4)	2 (33.3)
$75,001-$100,000	16 (9.4)	11 (12.4)	4 (7.4)	1 (3.6)	4 (3.5)	2 (2.9)	2 (6.3)	0 (0.0)
$100,001 or more	15 (8.8)	12 (13.5)	2 (3.7)	1 (3.6)	59 (51.8)	44 (63.8)	13 (40.6)	2 (33.3)
Employment								
Employed Full-Time	70 (40.9)	49 (55.1)	14 (25.9)	7 (25.0)	43 (37.7)	31 (43.7)	11 (34.4)	1 (16.7)
Employed Part-Time	39 (22.8)	20 (22.5)	12 (22.2)	7 (25.0)	10 (8.8)	8 (11.3)	2 (6.3)	0 (0.0)
Student	21 (12.3)	9 (10.1)	10 (18.5)	2 (7.1)	0 (0.0)	0 (0.0)	0 (0.0)	0 (0.0)
Retired	0 (0.0)	0 (0.0)	0 (0.0)	0 (0.0)	45 (39.5)	27 (38.0)	14 (43.8)	4 (66.7)
Medical Leave/Disability	9 (5.3)	0 (0.0)	2 (3.8)	6 (21.4)	8 (7.0)	4 (5.6)	2 (6.3)	2 (33.4)
Unemployed	32 (18.7)	10 (11.2)	15 (27.8)	6 (21.4)	7 (6.1)	4 (5.6)	3 (9.4)	0 (0.0)
Relationship Status								
Single	93 (54.4)	47 (52.8)	32 (59.3)	14 (50.0)	14 (12.3)	11 (15.5)	2 (6.3)	1 (16.7)
Committed/Partnered	50 (29.2)	26 (29.2)	16 (29.6)	8 (28.6)	5 (4.4)	3 (4.2)	1 (3.1)	1 (16.7)
Married	27 (15.8)	15 (16.9)	6 (11.1)	6 (21.4)	74 (64.9)	4 (5.6)	19 (59.4)	4 (66.7)
Divorced	1 (0.6)	1 (1.1)	0 (0.0)	0 (0.0)	11 (9.6)	2 (2.8)	7 (21.9)	0 (0.0)
Widowed	0 (0.0)	0 (0.0)	0 (0.0)	0 (0.0)	5 (4.4)	51 (71.8)	3 (9.4)	0 (0.0)
Have at least 1 child	32 (18.7)	15 (16.9)	13 (24.1)	4 (14.3)	59 (51.8)	36 (48.0)	20 (60.6)	3 (50.0)
Months since diagnosis (M, SD)	32.35 (19.25)	33.27 (11.47)	31.47 (28.27)	31.07 (18.60)	10.73 (34.22)	9.44 (23.25)	14.47 (53.39)	6.17 (6.68)

*Note*. Some categories may not total 100 percent due to missingness.

**Table 2:** DAI and cancer-related outcomes in young adults with testicular cancer.

	Job Problems				Family Problems				Sexual Problems				Treatment Side Effects			
	B	SE	*β*	95% CI	B	SE	*β*	95% CI	B	SE	*β*	*95% CI*	*B*	*SE*	*β*	*95% CI*
Block 1	Δ*R^2^* = .100				Δ*R^2^* = .034				Δ*R^2^* = .037				Δ*R^2^* = .053			
Age	−.03	.01	−.12*	−.06; .00	−.01	.02	−.02	−.04; .03	.02	.01	.08	−.01; .04	<.01	.01	.01	−.02; .02
Months since diagnosis	.01	<.01	.10	.00; .01	<.01	<.01	.03	−.01; .01	<.01	<.01	.07	<.00; .01	.00	<.01	.01	<.00, .00
Income	−.04	.03	−.09	−.09, .01	−.02	.04	−.03	−.09; .05	<−.01	.03	−.01	−.05; .05	<.00	.02	<.00	−.03; .03
Block 2	Δ*R^2^* = .429				Δ*R^2^* = .214				Δ*R^2^* = .182				Δ*R^2^* = .436			
DAI	3.69	.30	.69***	3.09, 4.28	2.93	.43	49***	2.08; 3.79	1.81	.31	.45	1.19; 2.42	2.17	.18	70***	1.80; 2.53

B = unstandardized beta; (β = standardized beta; CI confidence interval; DAI = Deficit Accumulation Index.

*Note*:

* *p*≤ .05;

*** *p* ≤ .001.

**Table 3:** DAI and HRQOL at 6 Months in adults with prostate cancer.

	Urinary Function				Bowel Function				Sexual Function				Hormonal Function			
	B	SE	*β*	95% CI	B	SE	*β*	95% CI	B	SE	*β*	*95% CI*	*B*	*SE*	*β*	*95% CI*
Block 1	Δ*R^2^* = .325				Δ*R^2^* = .554				Δ*R^2^* = .338				Δ*R^2^* =.476			
Age	.09	.25	.04	−.41; .60	−.01	.16	−.01	−.33; .30	−.56	.41	−.16*	−1.39; .27	−.27	.15	−.16	−.58; .04
Months since diagnosis	.01	.06	.01	−.12; .13	−.03	.04	−.07	−.11; .05	−.17	.12	−.18	−.39; .05	*<*.01	.04	.01	−.07; .08
Income	1.44	.66	.25*	.12; 2.76	.45	.42	.10	−.39; 1.30	.90	1.14	.09	−1.40; 3.19	.58	.41	.13	−.23; 1.39
Baseline function	.45	.13	.48***	.20; .71	.75	.10	.75***	.55; .94	.48	.13	.52***	.21; .74	.41	.10	.43***	.21; .61
Block 2	Δ*R^2^* = .001				Δ*R^2^* = .001				Δ*R^2^* = .001				Δ*R^2^* =.084			
DAI	−5.43	18.88	−.04	−43.26; 32.40	3.01	11.30	.03	−19.61; 25.63	19.98	32.74	.09	−45.85; 85.81	−38.88	11.89	−.35**	−62.69; −15.07

B = unstandardized beta; β = standardized beta; CI = confidence interval; DAI = Deficit Accumulation Index.

*Note*:

* *p*≤ .05;

*** *p* ≤ .001.

**Table 4:** Multiple regression analyses testing DAI and inflammation biomarkers at baseline.

	CRP				IL-6				sTNFαRII				IL-1β				IL-8			
	B	SE	*β*	95% CI	B	SE	*β*	95% CI	B	SE	*β*	*95% CI*	*B*	*SE*	*β*	*95% CI*	*B*	*SE*	*β*	*95% CI*
Block 1	Δ*R^2^* = .270				Δ*R^2^* = .076				Δ*R^2^* = .040				Δ*R^2^* = .476				Δ*R^2^* = .507			
Age	−.05	.04	−.20	−.13; .04	<.01	.02	.02	−.04; .06	.01	.01	.19	−.01; .03	.03	.03	.19	−.03; .10	.02	.01	.01	−.01; .05
Months since diagnosis	.01	.01	−.17	−.01; .03	<.01	.01	.11	−.01; .02	.00	<.01	.04	−.01; .01	<.00	.01	−.10	−.02; .01	<.00	<.01	.01	−.01; .01
Income	−.18	.08	−.36*	−.35; −.01	−.04	.05	−.13	−.14; .06	.02	.02	.19	−.02; .05	−.12	.08	−.31	−.28; .05	−.13	.03	−.67***	−.18; −.07
Block 2	Δ*R^2^* = .065				Δ*R^2^* = .258				Δ*R^2^* = .049				Δ*R^2^* = .084				Δ*R^2^* = .015			
DAI	3.97	2.45	.27	−1.05; 9.00	4.63	1.43	.54**	1.69; 7.57	.58	.49	.24	−.42; 1.58	2.87	1.82	.34	−.95; 6.68	.71	.77	.13	−.87; 2.29

B = unstandardized beta; β = standardized beta; CI = confidence interval; DAI = Deficit Accumulation Index; IL-6 = interleukin-6; CRP = C-reactive protein; sTNFαRII = soluble tumor necrosis factor receptor II; IL-1β = interleukin-1 beta; IL-8 = interleukin-8.

*Note*:

* *p*≤ .05;

*** *p* ≤ .001.

## Data Availability

The authors do not have permission to share data.

## Appendix A. Supplementary data

Supplementary data to this article can be found online at https://doi.org/10.1016/j.socscimed.2026.119332.
