# Supplementary material for: Aging-related deficits accumulation and cancer-related outcomes in testicular and prostate cancer: Cross-sectional and longitudinal findings
Source: Soc Sci Med. Author manuscript; Available in PMC 2026 May 24. (PMC13198736; doi:10.1016/j.socscimed.2026.119332)
Supplement: Supplementary Table 1 [file NIHMS2172835-supplement-Supplementary_Table_1.pdf]

Supplemental Table 1: Deficits Accumulation Index Items

DAI Construct	Study 1 Item	Study 2 Item	Value/Criteria	DAI Item Weight
1. Can take bath/shower		Does your health now limit you in bathing yourself?	Yes limited a lot	1
			Yes, limited a little	0.5
			No Not limited at all	0
			Missing	-
2. Can walk	Have you been physically limited as a result of your disease or treatment?	Does your heath now limit you in walking one block?	Yes limited a lot	1
			Yes, limited a little	0.5
			No Not limited at all	0
			Missing	-
3. Health limited moderate activities	During the past 4 weeks, have you had any of the following problems with your work or other regular daily activities as a result of your physical health: limited in moderate work or other activities?	Does your health now limit you in moderate activities, such as moving a table, pushing a vacuum cleaner, bowling, or playing golf?	Yes limited a lot	1
			Yes, limited a little	0.5
			No Not limited at all	0
			Missing	-
4. Can eat and dress		Does your health now limit you in dressing yourself?	Yes	1
			No	0
			Missing	-
5. Take care of oneself		Does your health now limit you in lifting or carrying groceries?	Yes	1
			No	0
			Missing	-
6. Trouble getting to bathroom on time		Overall, how big a problem has urinary urgency been for you during the last 4 weeks?	Yes, and the condition is still present	1
			No or Yes but the condition is no longer present	0
			Missing	-
7. Health limited climbing stairs	Does your health now limit you in climbing several flights of stairs?	Does your health limit you in these activities: Climbing several flights of stairs.	Yes limited a lot	1
			Yes, limited a little	0.5
			No Not limited at all	0
			Missing	-
8. Can go shopping for groceries or clothes		Does your health now limit you in lifting or carrying groceries?	Yes limited a lot	1
			Yes, limited a little	0.5
			No Not limited at all	0
			Missing	-

9. Can do your housework	Does your health limit you in these activities: Moderate activities, such as moving a table, pushing a vacuum cleaner, bowling, or playing golf?	During the past 4 weeks, how much did pain interfere with your normal work?	Quite a bit	0.75
			Moderately	0.5
			A little bit	0.25
			Not at all	0
			Missing	-
10. Pain	I have aches, pain, or discomfort.	How much bodily pain have you had during the past 4 weeks?	Very Severe	1
			Severe	0.8
			Moderate	0.6
			Mild	0.4
			Very Mild	0.2
			None	0
			Missing	-
11. General Health	In general, how would you say your health is?	In general, how would you say your health is?	Poor	1
			Fair	0.75
			Good	0.5
			Very Good	0.25
			Excellent	0
			Missing	-
12. Physical health limited work or activities	During the past 4 weeks, how much of the time have you had any of the following problems with your work or other regular daily activities as a result of your physical health: Cut down on the amount of time you spent on work or other activities	During the past 4 weeks as a result of your physical health did you cut down amount of time on the amount of time you spent on work or other activities?	All of the time	1
			Most of the time	0.75
			Some of the time	0.5
			A little of the time	0.25
			None of the time	0
			Missing	-
13. Emotional problems limited work or activities	During the past 4 weeks, have you had any of the following problems with your work or other regular daily activities as a result of any emotional problems (such as feeling depressed or anxious): Didn't do work or other activities as carefully as usual?	During the past 4 weeks as a result of your physical health did you cut down amount of time on the amount of time you spent on work or other activities?	All of the time	1
			Most of the time	0.75
			Some of the time	0.5
			A little of the time	0.25
			None of the time	0
			Missing	-
14. Physical or emotional health interfered with social activities	During the past 4 weeks, how much of the time has your physical health or emotional problems interfered with your social activities (like visiting with friends, relatives, etc.)?	During the past 4 weeks, to what extent has your physical or emotional problems interfered with your normal social activities?	Extremely	1
			Quite a bit	0.75
			Moderately	0.5
			Slightly	0.25
			Not at all	0
			Missing	-

15. Physical health resulted in accomplishing less	During the past 4 weeks, how much of the time have you had any of the following problems with your work or other regular daily activities as a result of your physical health?<br>Accomplished less than you would like to?	During the past 4 weeks as a result of your physical health did you accomplish less than you would like?	All of the time	1
			Most of the time	0.75
			Some of the time	0.5
			A little of the time	0.25
			None of the time	0
			Missing	-
16. Emotional problems resulted in accomplishing less	During the past 4 weeks, how much of the time have you had any of the following problems with your work or other regular daily activities as a result of your emotional health? Accomplished less than you would like to?	During the past 4 weeks as a result of your emotional health did you b) accomplish less than you would like......	All of the time	1
			Most of the time	0.75
			Some of the time	0.5
			A little of the time	0.25
			None of the time	0
			Missing	-
17. Had a lot of energy	How much of the time during the past 4 weeks did you have a lot of energy?	How much of the time during the past 4 weeks did you have a lot of energy?	All of the time	0
			Most of the time	0.25
			Some of the time	0.5
			A little of the time	0.75
			None of the time	1
			Missing	-
18. Felt downhearted or blue	How much of the time during the past 4 weeks have you felt downhearted and blue?	How much of the time during the past 4 weeks have you felt downhearted and blue?	All of the time	1
			Most of the time	0.75
			Some of the time	0.5
			A little of the time	0.25
			None of the time	0
			Missing	-
19. Felt calm or peaceful	How much of the time during the past 4 weeks have you felt calm and peaceful?	How much of the time during the past 4 weeks have you felt calm and peaceful?	All of the time	0
			Most of the time	0.25
			Some of the time	0.5
			A little of the time	0.75
			None of the time	1
			Missing	-
20. Heart Disease Comorbidity:	Do you have any of the following medical conditions: Peripheral vascular disease, (includes atherosclerosis, aortic aneurysm), etc.	Do you have any of the following medical conditions: Peripheral vascular disease (includes atherosclerosis, aortic aneurysm), etc.	Yes	1
			No	0
			Missing	-

21. Diabetes Comorbidity	Do you have any of the following medical conditions: Diabetes?	Do you have any of the following medical conditions: Diabetes?	Yes	1
			No	0
			Missing	-
			Yes	1
22. Respiratory Comorbidity	Do you have any of the following medical conditions: Chronic pulmonary disease (COPD, includes emphysema, asthma, chronic bronchitis, obstructive sleep apnea)?	Do you have any of the following medical conditions: Chronic pulmonary disease (COPD, includes emphysema, asthma, chronic bronchitis, obstructive sleep apnea)?	Yes	1
			No	0
			Missing	-
23. Chronic Liver, Kidney, or Gastrointestinal Comorbidity		Do you have any of the following medical conditions: Mild liver disease (includes hepatitis), constipation, or other gastrointestinal condition	Yes	1
			No	0
			Missing	-
24. Other Cancer/Leukemia Comorbidity	Do you have any of the following medical conditions: Cancer (excluding testicular cancer and non-melanoma skin cancer)?	Do you have any of the following medical conditions: Cancer (excluding prostate cancer and non-melanoma skin cancer)?	Yes	1
			No	0
			Missing	-
25. Glaucoma, Cataracts, or decreased vision Comorbidity	Have you ever been told by a doctor or other heath are professional you have or have had legal blindness, problems with double vision, or other eye problems?		Yes	1
			No	0
			Missing	-
26. Blood Pressure Comorbidity	Do you have any of the following medical conditions: Hypertension (high blood pressure)	Do you have any of the following medical conditions: Hypertension (high blood pressure)	Yes	1
			No	0
			Missing	-
27. Cerebrovascular Disease Comorbidity		Do you have any of the following medical conditions: Cerebrovascular disease	Yes	1
			No	0
			Missing	-
28. Thyroid Comorbidity		Do you have any of the following medical conditions: Thyroid condition	Yes	1
			No	0
			Missing	-
29. Shingles or other chronic infections	Do you have any of the following medical conditions: Infectious illness including HIV		Yes	1
			No	0
			Missing	-
30. Weight/BMI	Weight in pounds/current height. Formula: $(weight/height^2)) * 703$	Weight in pounds/current height. Formula: $(weight/height^2)) * 703$	Obese BMI >=30	1
			Underweight BMI <18.5	1
			Normal/Overweight BMI >=18.5 and <30	0

			Missing	-
31. Poly-pharmacy	Please list any prescription medications you have taken in the past 7 days	Please list any prescription medications you have taken in the past 7 days	>= 5 Prescription Meds	1
			<5 Prescription Meds	0
			Missing	-
32. Timed up and go			>6 seconds	1
			<=6 seconds	0
33. Depression	Brief Symptom Inventory Depression Subscale	Center for Epidemiologic Studies Depression Scale (CES-D)	T-Score <=65	0
			T-Score >65 to <=70	0.5
			T-Score >=70	1
			Missing	-
34. Anxiety	Brief Symptom Inventory Anxiety Subscale	General Anxiety Disorder - 7	T-Score <=65	0
			T-Score >65 to <=70	0.5
			T-Score >=70	1
			Missing	-
35. Fatigue	SF Vitality Subscale	SF Vitality Subscale	T-Score <10th percentile	1
			T-Score >=10th percentile	0
			Missing	-
36. Hearing Problems	I have problems with my hearing or have had "ringing" in my ears.		All of the time	1
			Most of the time	0.75
			Some of the time	0.5
			A little of the time	0.25
			None of the time	0
			Missing	-
37. Smell and taste problems	Have you had problems with your sense of taste or smell?		Yes limited a lot	1
			Yes, limited a little	0.5
			No Not limited at all	0
			Missing	-
38. Weakness	I lack energy.		All of the time	1
			Most of the time	0.75
			Some of the time	0.5
			A little of the time	0.25
			None of the time	0
			Missing	-
39. Los of sense of touch	Have you had tingling or numbness in your fingers or toes?		Yes limited a lot	1
			Yes, limited a little	0.5
			No Not limited at all	0
			Missing	-

40. Vigorous Activity	There are limits to my exercise/work-out because of my health	Does your health now limit you in vigorous activities, such as running, lifting heavy objects, participating in strenuous sports?	All of the time	1
			Most of the time	0.75
			Some of the time	0.5
			A little of the time	0.25
			None of the time	0
			Missing	-
41. Arthritis	Do you have any of the following medical conditions: Arthritis	Do you have any of the following medical conditions: Arthritis	Yes	1
			No	0
			Missing	-
